# Attenuation of mGluR1/5-dependent synaptic plasticity and ERK pathway dysfunction in the hippocampus of diabetic rats

**DOI:** 10.3389/fnins.2026.1814915

**Published:** 2026-04-01

**Authors:** Hayuma Otsuka, Sachie Sasaki-Hamada, Hitoshi Ishibashi, Jun-Ichiro Oka

**Affiliations:** 1 Laboratory of Pharmacology, Faculty of Pharmaceutical Sciences, Tokyo University of Science, Chiba, Japan; 2 Department of Physiology, School of Allied Health Sciences, Kitasato University, Kanagawa, Japan

**Keywords:** diabetes mellitus, electrophysiological experiment, hippocampus, long-term depression, mGluRs, rat

## Abstract

Streptozotocin-induced diabetic rats (STZ rats), an established animal model of type 1 diabetes mellitus, develop cognitive decline, which has been linked to impairments in hippocampal synaptic plasticity. Long-term depression (LTD) in the hippocampus may be induced by the activation of different types of G protein-coupled receptors, particularly metabotropic glutamate receptors (mGluRs) and muscarinic acetylcholine receptors. We previously demonstrated that acetylcholine receptor activation-dependent LTD was impaired in STZ rats, and herein investigated group I mGluR (mGluR1/5)-dependent LTD in the Schaffer collateral-CA1 synapses of STZ rats. Extracellular field recordings revealed that the chemical activation of mGluR1/5 with (S)-3,5-dihydroxyphenylglycine (DHPG, 50 μM, 10 min) induced sustained LTD in both control and STZ rats; however, the magnitude of DHPG-LTD was significantly smaller in STZ rats. Moreover, the paired-pulse ratio between before and 80 min after the application of DHPG increased in both control and STZ rats, and DHPG-LTD was independent of NMDA receptor activation. A Western blot analysis showed that DHPG-induced extracellular signal-regulated kinase (ERK) phosphorylation was reduced in STZ rats, whereas DHPG-induced phosphoinositide-dependent kinase 1 phosphorylation and the expression level of the scaffold protein, Homer1, were unchanged. Collectively, these results suggest that impaired ERK/MAPK signaling affected hippocampal mGluR1/5-dependent LTD in STZ rats, and the dysregulation of ERK may contribute to diabetes-associated cognitive decline because of its crucial role in protein synthesis-dependent synaptic plasticity.

## 1 Introduction

The number of cases of type 1 diabetes mellitus (T1DM), a chronic autoimmune disease, is increasing worldwide and affects millions of individuals across all age groups, not only children, but also adults due to decreased mortality (International Diabetes Federation, 2025). T1DM is characterized by chronic complications, including cardiovascular disease, diabetic retinopathy, and diabetic kidney disease. T1DM may also be accompanied by cognitive impairments in processing speed, attention, executive function, and memory (Chaytor, 2016).

Streptozotocin (STZ) is frequently used to chemically induce T1DM in animals. Cognitive decline develops in STZ-induced diabetic rats (STZ rats), and correlates with structural and functional deficits in brain areas, such as the hippocampus (Gaspar et al., 2016). Electrophysiological experiments showed that long-term potentiation (LTP) or long-term depression (LTD) was also impaired in STZ rats (Gispen and Biessels, 2000; Sasaki-Hamada et al., 2012). We previously demonstrated that the effects of STZ-induced diabetes on hippocampal synaptic plasticity, LTP, and LTD differed depending on the onset age (Sasaki-Hamada et al., 2012). Since LTD in the context of G protein-coupled receptors (GPCRs) remains unclear, the present study focused specifically on a young-adult onset STZ model, which has predominantly been used in research.

The slower and long-lasting effects of glutamate are generally mediated by metabotropic glutamate receptors (mGluRs), a family of GPCRs that modulate synaptic plasticity (Reiner and Levitz, 2018). Previous studies on STZ rats showed group I metabotropic glutamate receptor (mGluR1/5) expression and signaling pathways in the hippocampus. Gene expression studies revealed decreases in mGluR5 mRNA levels in the hippocampus of STZ rats (Balakrishnan et al., 2009). The prototypical mitogen-activated protein kinase (MAPK) family member, extracellular signal-regulated kinase (ERK) has been identified as an important kinase phosphorylating and regulating mGluR1/5 (Mao and Wang, 2016). However, previous studies reported that the level of ERK1/2 phosphorylation was higher (Dalli et al., 2018) and also unchanged (Jing et al., 2013) in the hippocampus of STZ rats. Nevertheless, mGluR1/5-dependent activity in STZ rats remain unclear.

Therefore, we examined mGluR1/5-dependent synaptic plasticity at hippocampal Schaffer collateral-CA1 (SC-CA1) synapses in STZ rats. We also showed mGluR1/5-dependent ERK activation in these rats.

## 2 Materials and methods

### 2.1 Chemicals

D-2-Amino-5-phosphonopentanoic acid (D-AP5) and (*S*)-3,5-dihydroxyphenylglycine (DHPG) were purchased from Tocris Cookson (Bristol, UK). STZ was purchased from Sigma-Aldrich (St. Louis, MO, USA). All other chemicals were purchased from FUJIFILM Wako Pure Chemical Industries (Osaka, Japan).

### 2.2 Animals

Animal experiments were performed in accordance with the requirements of the ARRIVE guidelines and the guidelines of the National Institute of Healthcare and Japan Neuroscience Society, and were approved by the Institutional Animal Care and Use Committee at Tokyo University of Science and Kitasato University. Male and female Wistar rats were obtained from Sankyo Labo Service Corporation and Nihon SLC (Shizuoka, Japan). T1DM was induced in 10-week-old rats by an intraperitoneal injection of STZ dissolved in phosphate-buffered saline (PBS) at a dose of 85 mg/kg.

Age-matched rats were administered an equivalent volume of PBS and used as controls. Prior to any experiments, the induction of DM was confirmed by measuring blood glucose levels using a glucose test meter (GUNZE Co., Kyoto, Japan). Animals with blood glucose levels >400 mg/dL were considered to be diabetic and, thus, were used in experiments. All animals were housed under controlled conditions with a 12-h light/dark cycle, a temperature of 23 ± 1 °C, and a relative humidity of 55 ± 5%, and were given *ad libitum* access to food and water.

### 2.3 Slice preparation

Hippocampal slices were prepared as previously described (Otsuka et al., 2024). The decapitation of 22- to 24-week-old rats was performed under isoflurane anesthesia. Using a vibratome (DTK-1000, Dosaka, Kyoto, Japan), 300- or 400-μm-thick acute transverse hippocampal slices were cut in ice-cold dissection buffer (in mM; 234 sucrose, 2.5 KCl, 1.25 NaH_2_PO_4_, 0.5 CaCl_2_, 10 MgSO_4_, 26 NaHCO_3_, and 11 D-glucose) saturated with 95% O_2_ and 5% CO_2_. The CA3 region was removed immediately after cutting to prevent the application of any chemicals from affecting CA1 neuronal activity through the stimulation of the CA3 region. Slices for electrophysiological recordings were recovered in artificial CSF (ACSF) (in mM; 124 NaCl, 3 KCl, 2.5 CaCl_2_, 1.3 MgSO_4_, 1.24 KH_2_PO_4_, 10 D-glucose, and 26 NaHCO_3_) at 36 °C for 50 min.

### 2.4 Extracellular field potential recordings

During electrophysiology recordings, 300-μm-thick slices were perfused with ACSF at a rate of 2–3 mL/min. The Schaffer collateral pathway was stimulated every 60 s using a bipolar tungsten electrode (WPI, Sarasota, FL, USA) to evoke extracellular field EPSPs (fEPSPs), which were recorded in the stratum radiatum of the hippocampal CA1 region using glass micropipettes (4–7 MΩ) filled with ACSF. The digital data of fEPSPs were recorded and analyzed as previously described (Otsuka et al., 2024). Test stimuli were delivered, a stable baseline at 60–70% of the maximum fEPSP amplitude was established, and 50 μM DHPG was then applied for 10 min to induce LTD. The significance of differences in the average fEPSP amplitude during the last 30 min of LTD was examined using the Student’s *t*-test.

A paired-pulse stimulation was delivered with a 30-ms inter-stimulus interval during the baseline period and 80 min after the application of DHPG. The paired-pulse facilitation (PPF) ratio was calculated by dividing the maximum negative slope of the second pulse by that of the first pulse. The slope was measured between 30 and 80% of the fEPSP peak amplitude.

### 2.5 Western blot analysis

Four hundred-micrometer-thick hippocampal slices (6 slices for each lane) were incubated in ACSF or 50 μM DHPG for 10 min and then homogenized in lysis buffer with a protease inhibitor cocktail (P8340, Sigma-Aldrich) and phosphatase inhibitor cocktails (P5726 and P0044, Sigma-Aldrich). Protein concentrations were measured using a BCA colorimetric protein assay (Thermo Fisher Scientific Inc., Rockford, IL, USA), and 40 μg of purified protein was resolved by SDS-PAGE and transferred onto a PVDF membrane. Regarding antibody detection, cells were incubated with the following primary antibodies diluted 1:1000 at 4 °C overnight: an anti-phospho-p44/42 MAPK (ERK1/2) Thr202/204 (#9101, Cell Signaling), anti-p44/42 MAPK (ERK1/2) (#4695, Cell Signaling), anti-phospho-PDK1 (Ser241) (#3061, Cell Signaling), or anti-PDK1 (#3062, Cell Signaling) antibody. Immunoreactive bands were then probed with a HRP-conjugated secondary antibody for 1 h, developed using the ECL detection system (Cytiva, Uppsala, Sweden), and visualized with FUSION SOLO 7S Edge (Vilber, Collégien France). The membranes were converted to digital images and protein expression was quantified by a densitometric analysis of each immunoreactive band using the Fusion-No system (18.06-dSN).

### 2.6 Data and statistical analyses

Data were analyzed and plotted using Igor Pro (WaveMetrics, OR, USA). Each plot or bar represents the mean ± S. E. M. Statistical analyses were conducted using GraphPad Prism 11 (Graphed software, San Diego, CA, USA) with the significance level set as *p* < 0.05. Data were checked for normality using a Shapiro–Wilk test. For normally distributed data, we used the Student’s *t*-test or a two-way ANOVA. ANOVA was used to examine the animal group and drug treatment. For non-normally distributed data, data were analyzed using the Mann–Whitney U test. An evaluation of PPF, based on comparisons between the second- and first-evoked potentials, was performed using the Student’s paired *t*-test or the Wilcoxon matched-pairs signed-rank test.

## 3 Results

### 3.1 Effects of T1DM on mGluR1/5-dependent LTD at hippocampal CA1 synaptic transmission

**Figure 1:**
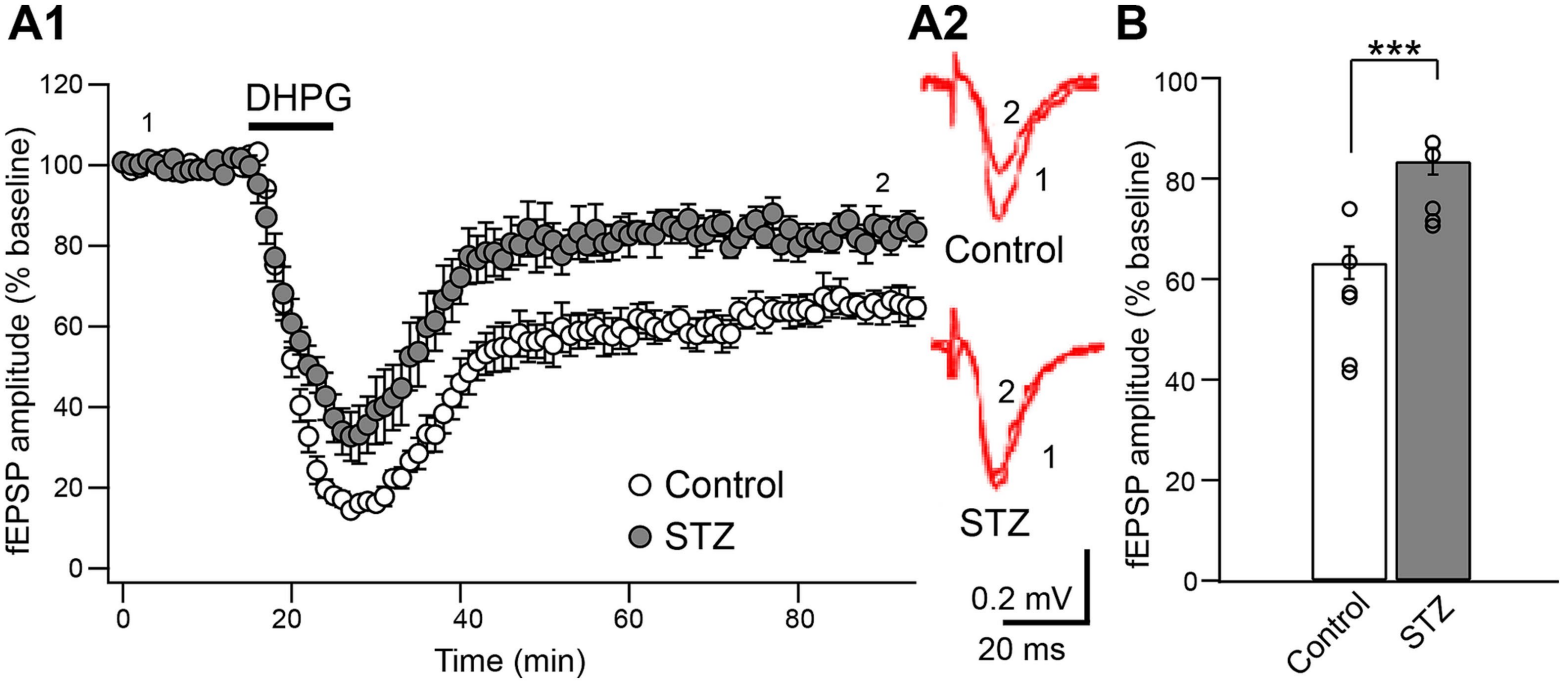
Attenuated DHPG-LTD at hippocampal SC-CA1 synapses in STZ rats. Hippocampal slices were obtained from control and STZ rats at 22–24 weeks. **(A1)** The bath application of the selective mGluR1/5 agonist, DHPG (50 μM, 10 min), to acute hippocampal slices induced LTD in control and STZ rats. **(A2)** Representative fEPSP traces at the time points indicated are shown in control (above) and STZ rats (bottom). **(B)** The bar graph shows the normalized fEPSP amplitude during the last 30 min of LTD induction, and the magnitude of DHPG-LTD was significantly attenuated in STZ rats compared with age-matched control rats (*n =* 6 in control, *n =* 7 in STZ; ***p < 0.001). Data are presented as the mean (± S. E. M).

We investigated whether the activation of mGluR1/5 by the selective agonist DHPG (50 μM, 10 min) induced LTD similarly in STZ rats and age-matched control rats (Figure 1A). The reduction in excitatory synaptic transmission in the CA1 region was sustained for 80 min after the washout of DHPG in both groups; however, the magnitude of DHPG-LTD during the last 30 min was significantly attenuated in STZ rats (control fEPSP: 63.2 ± 3.2% of the baseline, *n =* 6; STZ fEPSP: 83.5 ± 2.7% of the baseline, *n =* 5, *p* = 0.001, the Student’s *t*-test) (Figure 1B).

### 3.2 The presynaptic component of DHPG-LTD was not affected in STZ rats

**Figure 2:**
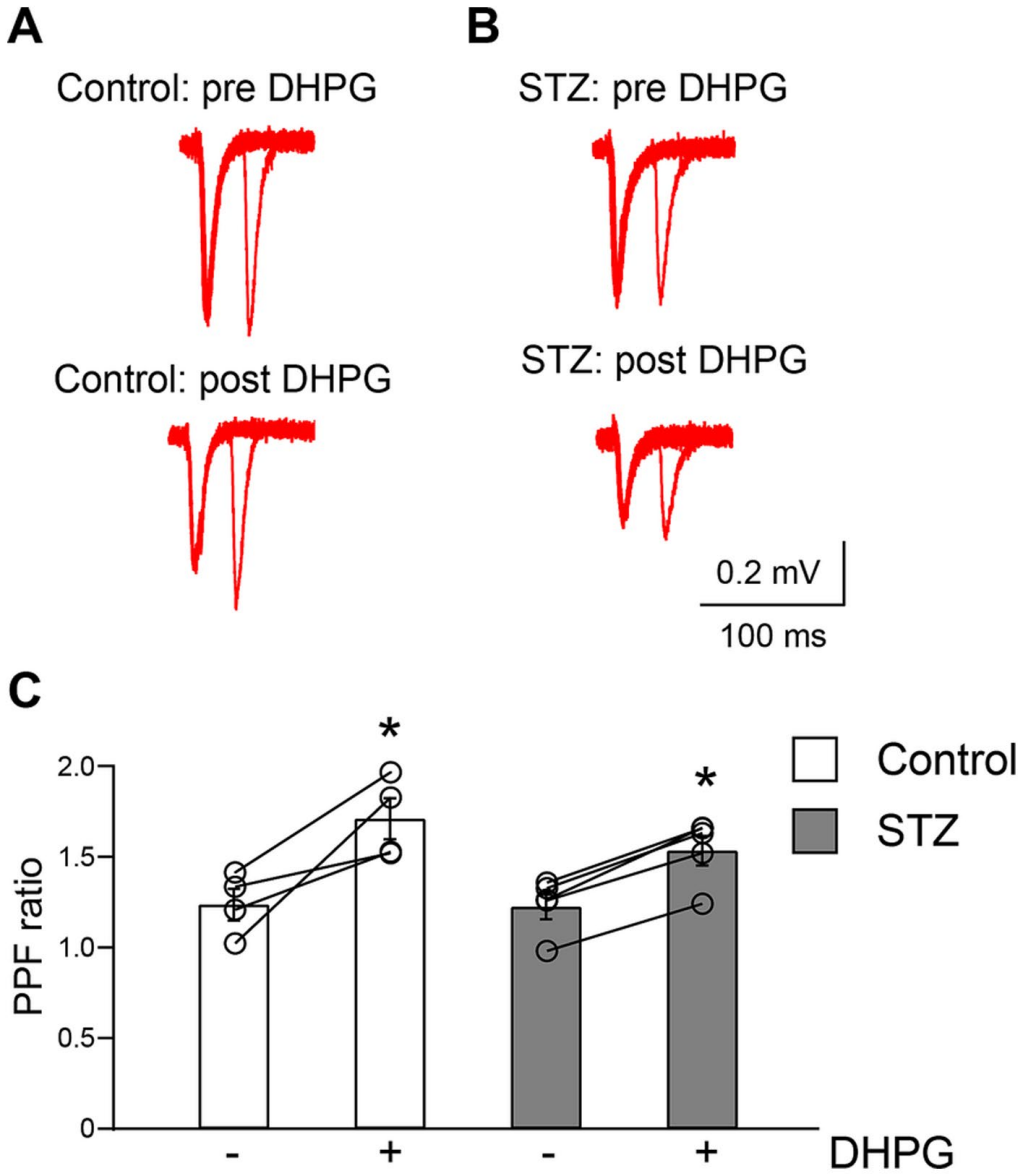
DHPG induced an increase in the PPF ratio in control and STZ rats. Inter-stimulus paired pulse stimulations were delivered in the baseline period (pre DHPG) and 80 min after the application of DHPG (post DHPG). Representative fEPSP traces in response to two consecutive stimulations in control **(A)** and STZ rats **(B)**. DHPG significantly affected the PPF ratio in control (*n =* 4 in control; *p < 0.05) and STZ rats (*n =* 5 in STZ; *p < 0.05) **(C)**. Data points represent averaged ratios (mean ± S. E. M) of the second to first responses to double stimuli at intervals of 30 ms.

To investigate the mechanisms underlying impaired DHPG-LTD in STZ rats, we compared the PPF ratio between control and STZ rats (Figure 2). The perfusion of DHPG was previously shown to induce an increase in the PPF ratio (Kumar and Foster, 2007; Tan et al., 2003). This finding revealed that the regulation of presynaptic neurotransmitter release was associated with DHPG-LTD. The paired-pulse stimulation was delivered with a 30-ms interval during the baseline period and 80 min after the washout of DHPG. The facilitation ratio significantly increased following the DHPG stimulation in both groups (control: *n =* 4, *p* = 0.02, the Student’s paired *t*-test; STZ: *n =* 5, *p* = 0.03, the Wilcoxon matched-pairs signed-rank test) (Figure 2C). These results indicate that the depression of presynaptic neurotransmitter release was sustained to a similar degree in both groups 80 min after the DHPG stimulation.

### 3.3 The activation of NMDA receptors (NMDARs) did not contribute to DHPG-LTD in STZ rats

**Figure 3:**
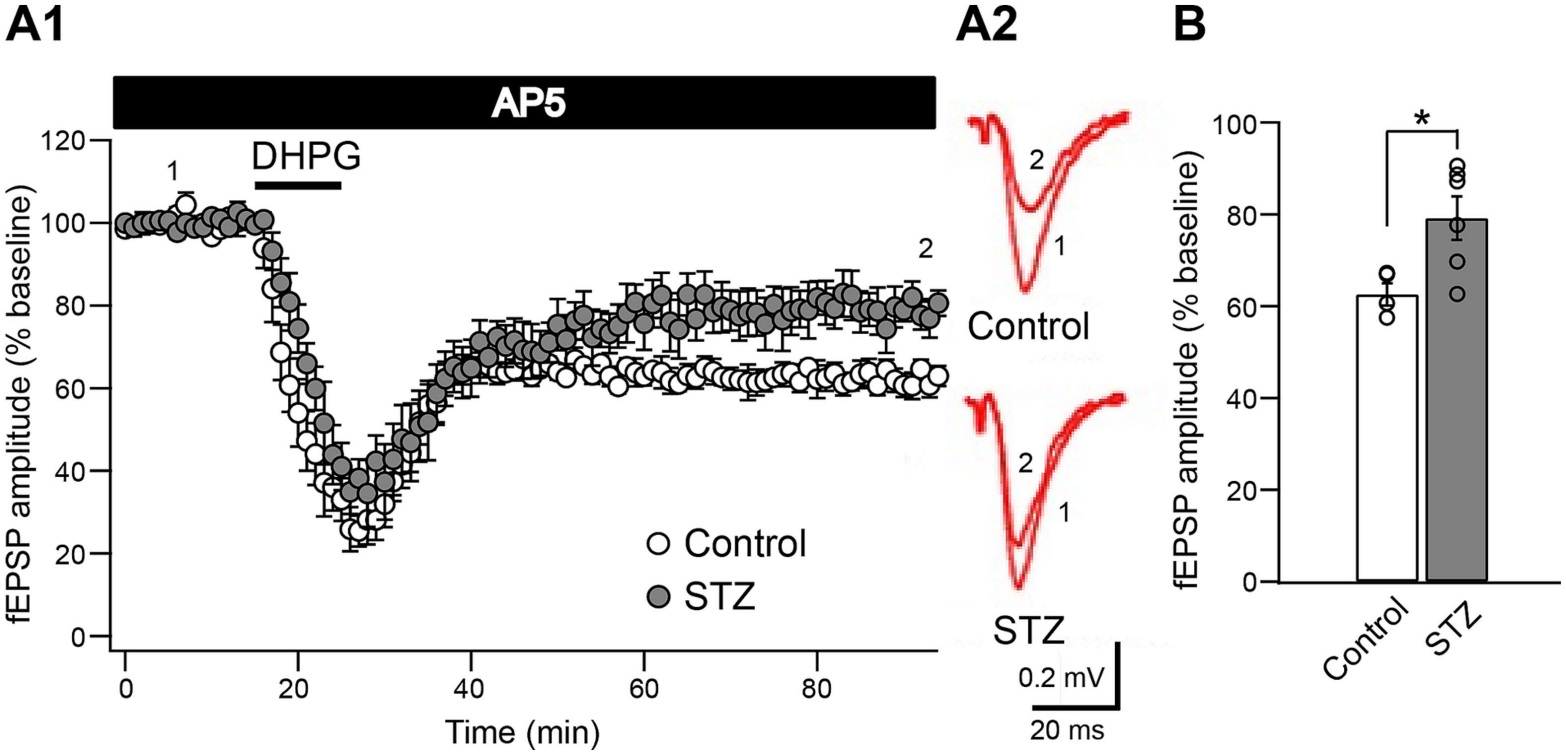
The synaptic co-activation of NMDA receptors was not required for the induction of DHPG-LTD in control and STZ rats. **(A1)** DHPG-LTD was not affected by the NMDA receptor antagonist, D-AP5 (50 μM), in control and STZ rats. **(A2)** Representative fEPSP traces at the time points indicated are shown in control (above) and STZ rats (bottom). (**B**) The bar graph shows the normalized fEPSP amplitude in the last 30 min of LTD induction (*n =* 4 in control, *n =* 6 in STZ; *p < 0.05). Data are presented as the mean (± S. E. M).

The application of DHPG has been shown to enhance the activity of NMDARs (Doherty et al., 2000; Fitzjohn et al., 1996; Harris et al., 2003; Mannaioni et al., 2001). Furthermore, the presence of an NMDAR antagonist inhibited DHPG-LTD in young adult rats, but not in aged rats (Kumar and Foster, 2007). To clarify whether impaired DHPG-LTD in STZ rats was attributed to the function of NMDARs, we examined DHPG-LTD under NMDAR blockade using the specific antagonist D-AP5 (50 μM) (Figure 3A). The magnitude of DHPG-LTD during the last 30 min was significantly smaller in STZ rats than in control rats (control: 62.5 ± 4.8% of baseline, *n =* 4; STZ: 79.2 ± 11.6% of baseline, *n =* 6; *p* = 0.02, the Student’s *t*-test) (Figure 3B). These results suggest that DHPG-LTD was induced independently of NMDAR activation in both groups.

### 3.4 The ERK pathway, but not the phosphoinositide 3-kinase (PI3K) pathway, was impaired in STZ rats

**Figure 4:**
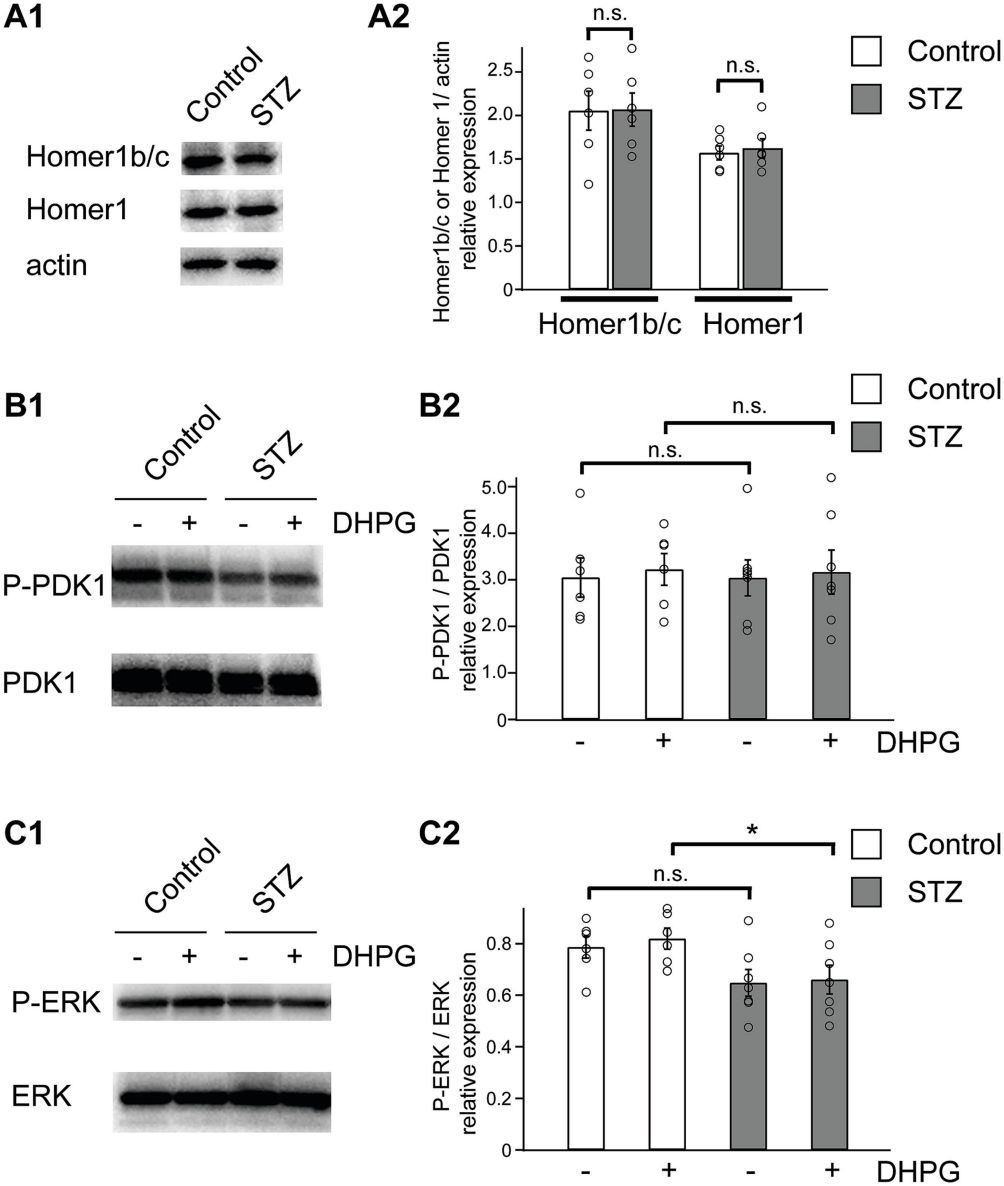
DHPG induced ERK activation in control rats, but not STZ rats. Representative western blots of Homer1b/c and total Homer1 expression between control and STZ rats **(A1)**. The bar graph shows the expression levels of Homer1b/c and total Homer1 proteins in the hippocampal CA1 region, normalized to actin **(A2)** (*n =* 6 in control, *n =* 6 in STZ). Representative western blots of DHPG-induced phosphorylation of the downstream effectors of PDK1 and ERK **(B1,C1)**. The bar graph shows the phosphorylation levels of PDK1 and ERK in the hippocampal CA1 region **(B2,C2)** (*n =* 6–7 in control, *n =* 6–7 in STZ). Data are presented as the mean (± S. E. M), and the significance of differences was set as *p < 0.05. n.s., not significant.

To further clarify the mechanisms underlying attenuated DHPG-LTD in STZ rats, we examined the postsynaptic scaffolding proteins and key signaling pathways implicated in DHPG-LTD expression. The postsynaptic scaffolding protein Homer1, which includes the isoforms Homer1b/c, has been shown to interact with mGluR1/5 and participate in the activation of downstream signaling pathways, thereby playing a critical role in DHPG-LTD (Hou and Klann, 2004; Ronesi and Huber, 2008). Accordingly, we examined the expression levels of Homer1b/c and total Homer1 proteins in the CA1 region. A Western blot analysis showed no significant differences in Homer1b/c or total Homer1 expression between control and STZ rats (control: Homer1b/c, 2.05 ± 0.22; Homer1, 1.57 ± 0.08, *n =* 6; STZ: Homer1b/c, 2.07 ± 0.19; Homer1, 1.62 ± 0.11, *n =* 6; values normalized to actin; *p* > 0.05, the Student’s *t*-test) (Figure 4A).

Since mGluR1/5 activate the PI3K-Akt-mammalian target of rapamycin (mTOR) and MAPK/ERK signaling pathways (Banko et al., 2006; Gallagher et al., 2004; Hou and Klann, 2004), we measured the relative levels of the phosphorylated forms of the PI3K effectors phosphoinositide-dependent kinase (PDK1) and ERK, which serve as representative downstream kinases of these pathways. Total protein samples from the CA1 region were prepared from slices immediately after the bath application of DHPG (50 μM, 10 min). DHPG-induced phosphorylation levels of PDK1 did not differ significantly between control and STZ rats (control: 3.22 ± 0.84, *n =* 6; STZ: 3.16 ± 1.25, *n =* 6; values normalized to total PDK1; *p* = 0.924, the Student’s *t*-test) (Figure 4B2). In contrast, DHPG-induced phosphorylation levels of ERK were significantly lower in STZ rats (control: 0.82 ± 0.10, *n =* 7; STZ: 0.66 ± 0.15, *n =* 7; values normalized to total ERK; *p* = 0.047, the Student’s *t*-test) (Figure 4C2). A two-way ANOVA showed a significant difference in the relative phosphorylation levels of ERK was observed between animal groups [*F*(1, 22) = 9.065, *p* = 0.006], but not between drug treatment [*F*(1, 22) = 0.208, *p* > 0.05]. These results provide no evidence for a change in the PI3K-PDK1 axis under the present experimental conditions. In contrast, the ERK pathway appeared to be selectively blunted in STZ rats.

## 4 Discussion

The present study demonstrated that the magnitude of chemically induced mGluR1/5-dependent LTD (DHPG-LTD) in the hippocampal CA1 region was significantly smaller in STZ rats than in age-matched controls (Figure 1). DHPG-LTD in both groups was accompanied by a similar increase in the PPF ratio (Figure 2), indicating that the contribution of reduced presynaptic transmitter release to LTD expression was equivalent between the groups, consistent with previous findings (Kumar and Foster, 2007; Tan et al., 2003). Based on these findings and the present results, attenuated DHPG-LTD in STZ rats was more likely to be attributed to postsynaptic rather than presynaptic mechanisms.

DHPG-LTD has been reported to occur independently of NMDAR activation from neonatal to adult rats (Kumar and Foster, 2007, 2014; Nosyreva and Huber, 2005; Tan et al., 2003). However, in aged rats, the NMDA-receptor antagonist significantly reduced, but did not completely eliminate DHPG-LTD, suggesting that the mechanisms underlying DHPG-LTD change with aging (Kumar and Foster, 2007, 2014). Here, we demonstrated that DHPG-LTD was induced independently of NMDAR activation in both control and STZ rats (22–24 weeks old) (Figure 3). These results suggest that NMDAR activation was unlikely to be the cause of attenuated DHPG-LTD under diabetic conditions. Furthermore, these results were consistent with our previous findings showing no significant difference in NMDAR activation between control and STZ rats (Otsuka et al., 2024; Sasaki-Hamada et al., 2012). Therefore, we investigated downstream mGluR1/5 receptors.

The synaptic scaffolding coiled-coil forms of Homer1, which include Homer1b/c, link membrane-bound mGluR1/5 receptors to downstream signaling molecules (Kammermeier, 2008; Lv et al., 2014; Tu et al., 1998). Previous studies demonstrated that hippocampal Homer1 or Homer1b/c was required for DHPG-LTD (Gimse et al., 2018; Ronesi and Huber, 2008). In non-obese diabetic mice, a polygenic model of T1DM, Homer1 protein expression was reduced in the hippocampus (Yokokawa et al., 2023). Therefore, we examined the protein expression of Homer1 and Homer1b/c in the hippocampus, and found no significant differences between control and STZ rats (Figure 4A).

Group I mGluRs (mGluR1/5) engage in multiple intracellular cascades, of which the PI3K-Akt–mTOR and ERK/MAPK pathways are recognized as key contributors to DHPG-LTD. Indeed, pharmacological inhibition of the ERK/MAPK pathway suppresses DHPG-LTD in hippocampal CA1 neurons (Gallagher et al., 2004). Consistent with this, mGluR-dependent LTD involves signaling pathways regulating synaptic protein synthesis, including ERK/MAPK and PI3K–mTOR pathways (Antion et al., 2008; Banko et al., 2006; Hou and Klann, 2004). In addition, alterations in ERK signaling have been reported in the hippocampus under diabetic conditions (Liu et al., 2019). Moreover, metabolic disturbances associated with diabetes, including hyperglycemia, oxidative stress, and impaired insulin signaling, have been proposed to influence ERK-dependent signaling pathways in neurons (Stranahan et al., 2008; Duarte et al., 2012).

DHPG-LTD is known to be modulated by phosphorylation mechanisms; for example, DHPG-LTD is impaired in calpain-1 knockout mice and can be rescued by phosphatase inhibitors (Zhu et al., 2017). Therefore, we evaluated the phosphorylation status using Western blot analysis in the current study. Our results demonstrated that DHPG-induced ERK phosphorylation, but not PDK1 phosphorylation, was significantly reduced in STZ rats (Figures 4B,C). Given the established role of ERK signaling in synaptic plasticity and its involvement in protein synthesis–dependent DHPG-LTD, these findings raise the possibility that impaired ERK signaling contributes to the attenuation of mGluR1/5-dependent LTD observed in STZ rats. Although we observed reduced ERK phosphorylation in STZ rats, the causal relationship between ERK signaling and impaired DHPG-LTD was not directly examined. Pharmacological or genetic manipulation of ERK signaling will be necessary to determine whether ERK dysfunction directly contributes to LTD impairment under diabetic conditions.

STZ rats have been reported to exhibit deficits in spatial learning, typically attributed to impairments in hippocampal LTP (Biessels et al., 1996; Kamal et al., 2000; Sasaki-Hamada et al., 2012). The present results extend this perspective by showing that DHPG-LTD was also attenuated in the CA1 region of STZ rats. mGluR1/5-dependent LTD is a physiologically relevant form of synaptic plasticity (Anwyl, 1999; Bellone et al., 2008), and attenuated mGluR1/5-dependent LTD is associated with deficits in the acquisition/consolidation of spatial learning and poor performance during task reversal (Privitera et al., 2019; Wall et al., 2018). Therefore, in addition to impaired LTP, attenuated mGluR1/5-dependent LTD may contribute to the spatial learning deficits observed in STZ rats. Thus, further studies combining behavioral analyses with molecular manipulations will be necessary to clarify the specific contribution of mGluR-dependent LTD to memory impairment of STZ rats.

Apart from these experimental findings, clinical studies have reported that patients with diabetes frequently exhibit cognitive dysfunction in type 1 and type 2 diabetes share many similarities, but important differences do exist (Lacy et al., 2022; McCrimmon et al., 2012). Therefore, future studies should investigate differences in cognitive impairment between type 1 and type 2 diabetes using not only the STZ model but also other models, such as db/db mice.

## Data Availability

The datasets presented in this study can be found in online repositories. The names of the repository/repositories and accession number(s) can be found in the article/supplementary material.
